# Transactions T. S. M. A., 1885

**Published:** 1885-10

**Authors:** 


### Bibliography.

Transactions Texas State Medical Association, 1885, a handsome octavo volume of 435 pages, in cloth and gold, red edges; printed on extra heavy S. and S. C cream book paper, by Draughon & Lambert, Austin, Texas. The binding, which is highly creditable, being done in Austin, by John Southgate. Issued by order of the Texas State Medical Association; Publishing Committee, Dr. F. E. Daniel, chairman; Dr. W. J. Burt, and Dr. W. B. Brooks.

This is about as neat a volume as we have seen emanating from any State Medical association. The contents consists of the officers for the year, the standing and other committees, the officers of the several sections, and the minutes of four days proceedings. The appendix, which is by far the most voluminous part, is made up of the President's address—an excellent paper by Dr. H. C. Ghent, of Belton, and the reports of the chairmen of sections, together with a large number of original papers read in each section, the whole number of those papers being about forty. They show a surprising amount of original research and observation of diseases common to this State, as modified by climate and surroundings; and taken altogether they are of a very fair order of excellence—better, perhaps, than those of the year previous. We think the amount and quality of scientific work done at the Houston meeting by this society will compare favorably with that of any other State, especially when we consider that Texas is comparatively a new State;—and her people mostly impoverished by the war, doctors as well as others—the members of the medical profession as a rule have had to work too hard to spare much time in recording their observations, and many have not been able to keep up with the current literature of the day, nor buy the standard works, nor supply themselves with the modern means of accurate diagnosis, and improved facilities for treatment, etc., until within the last few years.

As we have not room to analyse or review all the papers, it might be thought invidious were we to particularize or single out one or more for discussion. Where all, or most all, are good, it would hardly be fair to make distinctions. Many of the papers will constitute valuable additions to the literature of the State.

---

Other Book Notices omitted for want of space.

